# Supplementary material for: Diabetic Complication Profiles and Associated Risk Factors: A Comprehensive Analysis from Two Public Hospitals in the Najran Region, Southern Saudi Arabia
Source: Medicina (Kaunas). 2025 Oct 18;61(10):1871. doi: 10.3390/medicina61101871 (PMC12566243; doi:10.3390/medicina61101871)
Supplement: Supplementary file 1 [file medicina-61-01871-s001.zip › medicina-3900413-supplementary.pdf]

Table S1. Variance Inflation Factor (VIF) analysis.

Variable	VIF	Multicollinearity
Diabetes type	28.34	Severe
BMI	8.57	Moderate
Treatment of diabetes	7.71	Moderate
Glycemic control	6.09	Moderate
Duration 2	4.21	Low
HbA1c	3.51	Low
Weight	3.30	Low
Occupation	2.49	Low
Sex	2.03	Low
Family history of diabetes	2.01	Low
Residence	1.89	Low
Age	1.49	Low
Hypertension	1.28	Low

Interpretation Scale

- **Severe multicollinearity**: VIF > 10.
- **Moderate multicollinearity**: VIF between 5 and 10.
- **Low multicollinearity**: VIF < 5.

Table S2. False Discovery Rate (FDR) correction using the Benjamin–Hochberg procedure.

Table	Significant Before FDR	Significant After FDR	Change
Table 1	14	13	−1
Table 2	1	1	0
Table 3	1	1	0
Table 4	1	0	−1
**Total**	**17**	**15**	**−2**

Summary

- **Total p-values tested**: 52
- **Significant before FDR (p < 0.05)**: 20
- **Significant after FDR (FDR < 0.05)**: 16
- **Change in significance**: −4
